# Does Regular Practice with a “Flexible Bronchoscopy Simulator” Improve Fibreoptic Intubation Skills in Experts and Novices? A Randomized Controlled Study

**DOI:** 10.3390/jcm12165195

**Published:** 2023-08-09

**Authors:** Maartje van Haperen, Tom C. P. M. Kemper, Jeroen Hermanides, Susanne Eberl, Markus W. Hollmann, Jennifer S. Breel, Benedikt Preckel

**Affiliations:** Department of Anaesthesiology, Amsterdam University Medical Centres, Location AMC, 1105AZ Amsterdam, The Netherlands; m.vanhaperen@amsterdamumc.nl (M.v.H.); t.kemper@amsterdamumc.nl (T.C.P.M.K.); j.hermanides@amsterdamumc.nl (J.H.); s.eberl@amsterdamumc.nl (S.E.); m.w.hollmann@amsterdamumc.nl (M.W.H.); j.s.breel@amsterdamumc.nl (J.S.B.)

**Keywords:** education, airway management, fibreoptic intubation, airway simulator, simulated difficult airway

## Abstract

Background: The appropriate management of a “difficult airway” remains a challenge for novices and experienced anaesthetists. With the current available airway technologies, e.g., video laryngoscopy, flexible bronchoscopy (fibreoptic intubation (FOI)) for endotracheal intubation is decreasing, likely diminishing caregiver skills. We investigated whether bronchoscopy simulator training improved FOI skills. Methods: 72 volunteers, consisting of anaesthetists, anaesthesia residents, and nurses, performed six exercises on a bronchoscopy simulator. At baseline and after 12 months, the six exercises included one serious game (to train agility), two basic airways, and three difficult airways. After a baseline assessment, subjects were randomly allocated to the intervention group (with) or control group without bronchoscopy simulator training every six weeks for 10 min using a preloaded serious game. The primary outcome was the difference in the time to reach the carina after 12 months, as measured objectively by the simulator. The level of stress and FOI confidence after 12 months were secondary outcomes. Results: The control and intervention groups had a similar time to reach the carina in difficult airway cases and the reported stress levels, at baseline and 12 months, showed no difference. In contrast, the intervention group’s self-reported confidence in FOI skills improved more. Conclusions: Although participants rated higher in confidence, practicing FOI skills on an airway simulator with an agility game did not increase their performance in simulated challenging airway instances.

## 1. Introduction

A difficult airway remains challenging, not only for novices, but also for experienced anaesthetists. Awake fibreoptic intubation (awake FOI) is an advanced technique for airway management that is frequently performed in patients with expected difficult airways. Awake FOI, together with video laryngoscopy, is still considered one of the two standard techniques for patients with an expected difficult airway [1,2]. However, exposure to an awake FOI has always been limited, and has decreased further during the last years partly due to the availability of supraglottic airway devices and video laryngoscopy [3,4]. In addition, the increased focus on patient safety along with the recommendation that the most experienced caregivers should perform an FOI also contributes to the decline in FOI exposure for some staff [1,5,6]. Previous studies have showed a wide range for the number of FOI, between ten and a hundred procedures, necessary to become competent in successfully and safely performing an FOI [7,8,9,10,11]. However, due to this limited exposure to awake FOI, it is difficult for residents to gain sufficient practice in obtaining the necessary FOI skills. Therefore, to ensure that anaesthetists and anaesthesia residents gain sufficient practice and maintain their level of proficiency with awake FOI, new educational strategies are necessary.

The rapid advances in computer science and bioengineering have facilitated new educational methods to train students in the respective situations [12,13,14]. Several studies have shown that practice on normal airways with “low fidelity” simulators and virtual reality leads to improved FOI skills [10,12,13,14,15,16,17,18]. However, until recently it was not possible to train on different abnormal airways, as there were no simulators to mimic the great variety of the abnormal airway. The ORSIM^®^ bronchoscopy simulator (ORSIM^®^ Airway Simulation Ltd., Auckland, New Zealand) is a combined high-fidelity and virtual reality simulation tool consisting of three components: a replica bronchoscope, an interface, and a laptop. These three components interact with the software to create a high-fidelity virtual reality simulation of normal and difficult airways. In 2016, Baker et al. showed that this simulator could be used to differentiate between various levels of competency and the FOI [14].

It is unknown whether repeated practice with a bronchoscopy simulator also improves awake FOI skills in clinical practice. We hypothesized that regular practice with a bronchoscopy simulator would improve agility and FOI skills, as measured by the time to successful visualization or reaching the carina during FOI. Additionally, we examined the relationship between participants’ level of awake FOI expertise and the time to successfully reach the carina during a simulated FOI.

## 2. Materials and Methods

### 2.1. Study Design

In this single-centre, prospective, randomized controlled study, we compared regular practice with the the ORSIM^®^ bronchoscopy simulator (Airway Simulation Limited, Auckland, New Zealand) (intervention group) and no additional training (control group) to maintain or even improve awake FOI skills. This study was conducted at the Amsterdam University Medical Centre (Amsterdam UMC) from March 2018 to July 2019.

### 2.2. Ethics

After reviewing the trial protocol, ethical approval for this study (Ethical Committee N° NAC 207) was provided by the Medical Ethics Review Committee of the Amsterdam UMC hospital, Amsterdam, The Netherlands (Secretary Dr. Y.E. Donselaar) on six March 2018. The Medical Ethics Review Committee of the Amsterdam UMC provided a waiver under the Medical Research Involving Human Subjects Act (W18_068#18.008). Written informed consent was obtained from all the participants. The study was registered in the ISRCTN registry (ISRCTN35432230) and conducted in compliance with the Declaration of Helsinki (Fortaleza, 2013). We adhered to the CONSORT guidelines for writing this report [19].

### 2.3. Participants

All participant anaesthetists, anaesthesia residents, or anaesthesia nurses were members of the anaesthesia department of the Amsterdam University Medical Centre location AMC, a tertiary teaching hospital, and were approached to participate on a voluntary basis. The only exclusion criterion was previous experience with an ORSIM^®^ bronchoscopy simulator. Participants were randomly assigned to one of two groups; the intervention group received regular practice in the use and agility of the fiberscope using a serious game “whack a mole” for ten minutes every six weeks; the control group received no additional simulator practice. Both groups were further divided into three equal subgroups, stratified by their level of experience in FOI, with anaesthetists regarded as experts, anaesthesia residents as trainees, and anaesthesia nurses as novices.

**Figure 1:**
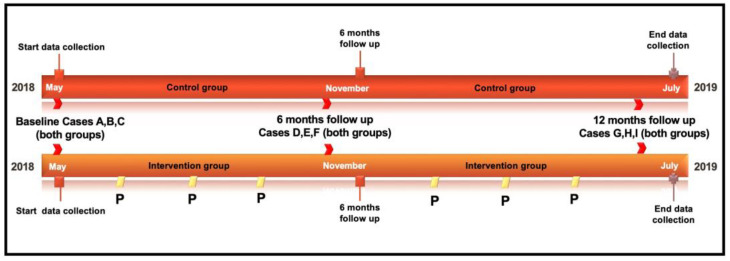
Timeline of the study protocol. P = ten minutes of practice for the intervention group with serious game “whack a mole”.

Participants in both groups were evaluated by performing three simulated ‘difficult airway’ cases on the bronchoscopy simulator, 6 months and 12 months after the baseline measurement (Figure 1).

The measurements were performed in a simulation laboratory in the presence of a single observer who assisted with the study setup and timing of the cases. A total of 72 participants were included between March and June 2018 and were followed up for a year until July 2019.

### 2.4. Simulation Training and Variables

At baseline and before randomization, participants received standardized slide and video training on how to use the bronchoscopy simulator. No additional instructions were given regarding the upper airway anatomy or how to handle the flexible bronchoscope during FOI. At each measurement (baseline, six months, and 12 months), the participants had time to familiarize themselves with the bronchoscopy simulator. This familiarization consisted of performing the digital “whack-a-mole” game and one simulated intubation case with a normal oral and nasal airway. At the end of the familiarization phase, all participants practiced three difficult intubation scenarios. These scenarios were designed to simulate various pathologies of the upper airway, and were intended to become increasingly difficult. To optimally mimic the clinical setting and to reduce pattern recognition, different cases were deliberately chosen, as well as the possibility of participants choosing between the oral or nasal insertion of the scope. With an awake FOI often being performed under limited time for patient comfort, participants from both groups had a maximum of ten minutes per case to successfully reach the carina. If they were unable to visualize the carina within ten minutes, the simulation was stopped.

The intervention consisted of practicing bronchoscopy every six weeks for a ten-minute practice by playing a digital “whack-a-mole” game. This game trained the participants in agility using a flexible bronchoscope. During the game, participants had to whack the gophers who popped up randomly at one of the twelve different holes. There were three different levels, with varying depths of the gophers within the holes. At the end of the game, the participants received a score (in percentages) based on their reaction speed and agility.

A questionnaire was used to determine the level and quantity of experience, as well as previous experience with FOI training. Participants were also asked to score their own ability and report their stress level when performing an awake FOI using a visual analogue scale (VAS) of 100 mm. A low VAS score indicates a low ability and low stress level when performing aFOI, a high score indicates good ability, and a high stress level indicates an awake FOI.

### 2.5. Data Sources and Measurements

The primary outcome variable was the time to successful intubation (in seconds), defined as the time from the introduction of the fiberscope to reaching the carina (ready for intubation). The start time of the clock was automatically registered using a bronchoscopy simulator.

### 2.6. Randomisation

We aimed to include 36 participants per group, with three equal subgroups of 12 participants per subgroup, stratified according to their level of seniority/experience in FOI. Simple randomization was performed using three sealed envelopes (one per seniority level). Each envelope contained paper strips with either C for the control or I for the intervention, which assigned the participant to either the control or intervention group.

### 2.7. Blinding

The person who assisted the participants with the study setup and timing was blinded to the group allocation.

### 2.8. Sample Size

The sample size calculation was based on a publication by Ost et al., who demonstrated that an improvement in speed of 50% can be expected with training [10]. According to Baker et al. [14], this improvement in speed is the smallest for experts who have an objective scenario time of 116 s (SD 17) without training. Theoretically, their scenario time would decrease (improvement in performance) to 58 s with training. To detect an improvement in the scenario time of at least 10% with training (i.e., an improvement from 116 to 104 s), we calculated a sample size of 33 participants per group, with a total of 66 participants. To correct for potential dropouts, 72 participants were included.

### 2.9. Statistical Analysis

All study data were stored in the ORSIM^®^ bronchoscopy simulator and then transferred to a Good Clinical Practice compliant database (Castor EDC) [20]. The quality of the data was ensured by double data entry, and all analyses were performed using IBM SPSS Statistics for Windows, version 26.0 (IBM Corp).

A *p*-value < 0.05 was considered statistically significant in two-tailed testing. Data are presented as mean ± standard deviation (SD) or median with interquartile range (IQR), depending on the distribution of the data. Categorical data are expressed as percentages and counts. To compare the time to successfully reach the carina between the control and intervention groups, we used the Mann–Whitney U test and performed a Kaplan–Meier analysis. For other secondary outcomes, such as the participants’ estimation of their own skills, reported stress level, and degree of training in performing an FOI, we used a Wilcoxon signed-rank test to assess between-group differences.

## 3. Results

A total of 72 participants, 24 per level of seniority, were included and subdivided into intervention and control groups. At 12 months, data from 94% (*n* = 68) of the participants were available for analysis. Baseline characteristics, including age, sex, previous experience with FOI, and background training, were not different between the groups (Table 1).

**Figure 2:**
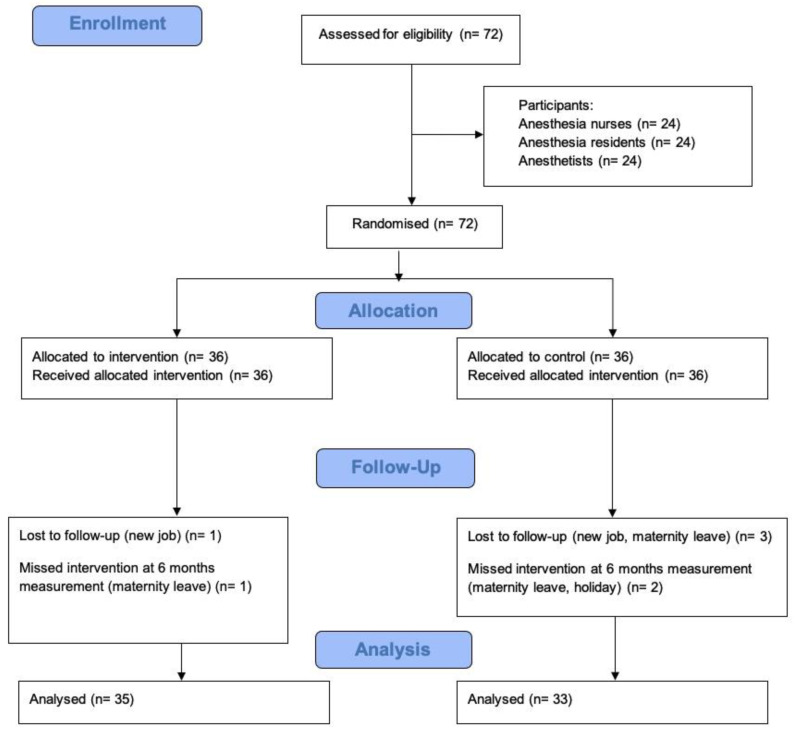
Flowchart of the study and data analysis.

Only participants with missing data at the 12-month time point were excluded from further analyses. There were two dropouts in each group after 12 months. Additionally, two participants in the control group discontinued the measurement at six months but were present after 12 months (Figure 2).

**Figure 3:**
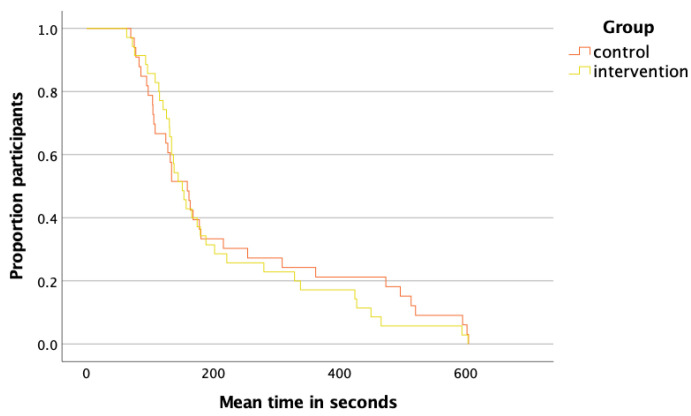
Kaplan–Meier curve for the time to reach the carina (in seconds) stratified by groups (time limit was set to 10 min).

Our results showed a median (IQR) time to reach the carina of an IQR 160 [105 to 336] seconds per total of six cases in the control group, and an IQR 152 [121 to 281] seconds in the intervention group. A Mann–Whitney U test (c_ontrol_ = 33, i_ntervention_ = 35); U = 573.5, z = −0.49, *p* = 0.96) did not show a significant between-group difference in the time to reach the carina. In addition, we used Kaplan–Meier analysis (Figure 3).

**Figure A1:**
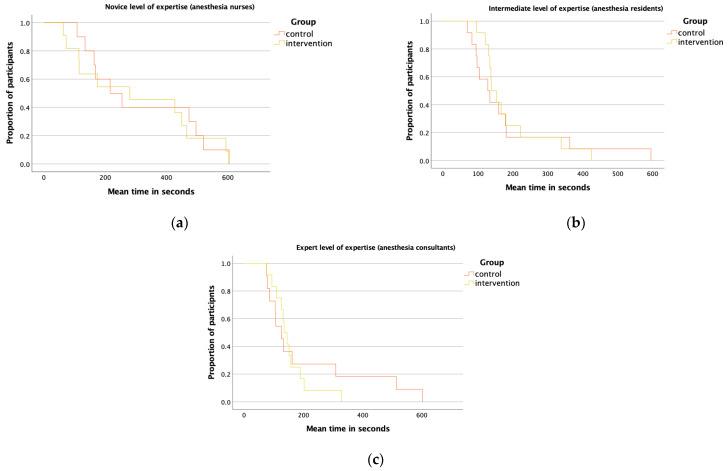
(**a**) Kaplan–Meier curve for the time to reach the carina (in seconds) in the group of anaesthesia nurses (time limit was set to 10 min), (**b**) Kaplan–Meier curve for the time to reach the carina (in seconds) in the group of anaesthesia residents (time limit was set to 10 min), and (**c**) Kaplan–Meier curve for the time to reach the carina (in seconds) in the group of anaesthesia consultants (time limit was set to 10 min).

The LR χ^2^ test for the difference in duration until successful visualization of the carina between the control and intervention groups showed a *p*-value of 0.572. Even after stratifying for the level of seniority, we did not observe a statistically significant difference between the groups (with overall comparisons χ^2^(1, *n* = 68) = 0.114, *p* =0.736 (Appendix A, Figure A1).

The Wilcoxon signed-rank test was used to assess the between-group differences in the participants’ estimation of their own skills and the level of stress using a VAS score. The participants’ assessment of their own skills improved in both groups between baseline and the end of the study, but showed no significant differences between the groups (U = 632.0, *p* = 0.373). The level of stress showed a trend towards a decrease in both groups between baseline and 12-month measurements (U = 602.5, *p* = 0.602) (Table 2).

Although more participants felt that they were sufficiently trained after 12 months (Table 2), a chi-square test of independence showed that there was no statistically significant difference between the intervention and control groups for the feeling of being sufficiently trained in the FOI (χ^2^(1, *n* = 68) = 1.89, *p* = 0.17).

## 4. Discussion

We found that regular practice with the ORSIM^®^ bronchoscopy simulator did not improve FOI skills in difficult airway cases, even when stratified by the level of seniority or the total amount of awake FOIs previously performed.

Similar to the studies of Wong et al. and Melvin et al., we compared training using the ORSIM^®^ simulator with conventional didactic training to improve FOI skills [17,18]. In contrast to these previous studies, our findings did not show a beneficial effect. One possible explanation for this discrepancy could be the choice to measure the training effect. In our study, the effect of training was objectively measured (using bronchoscopy simulator scores), whereas previous studies assessed the effect of training more subjectively (based on videos of performance in a clinical setting, assessed by independent assessors) [9,17,18,21,22,23]. In addition, these previous studies and the one by Cailleau et al. demonstrated the effect of training on airways with a normal anatomical structure [24], whilst our investigation that focused on difficult airways showed no improvement. However, this is in accordance with the study by Cailleau et al. To best simulate the clinical setting and limit the impact of learning on individual cases, each was completed only once. Therefore we also did not provide additional guidance on FOI or airway anatomy.

Our decision to score performance solely on the bronchoscopy simulator was influenced by the low incidence of difficult airway situations requiring an awake FOI in the clinical setting [1,3,25]. We did not intend to potentially distract or induce stress in colleagues by evaluating their performance during an awake FOI in a patient with a known ‘difficult airway’ in real-life cases.

We included a six-week time interval between the last practice on the bronchoscopy simulator and the performance assessment, whereas other studies assessed performance directly or within a week after training. Therefore, it is possible that the lack of any training effect in our study was based on a training effect that weans off after some time [1,9,12,13,14,26,27,28,29]. This supposed weaning-off effect of the training needs to be considered in future studies investigating training effects on technical skills.

Previous studies have showed skill improvement with varying practice strategies, and the duration of, and the time interval between practices, as well as the types of practices given [16,22,23,24,30]. Further research should focus on designing an evidence-based training system for teaching and assessing the performance of an awake FOI, with a focus on recognising the altered airway anatomy.

### Limitations and Strength of the Study

Our study was limited by the choice to practice with the intervention group every six weeks on the game “whack-a-mole,” as we did not explore whether practicing more frequently, for a longer period, or utilizing normal airway scenarios instead of a serious game for training would have further improved the training results. However, previous studies by Naik et al. and Melvin et al. also used a game “choose-the-hole” to improve FOI skills, with a difference of 45 min in practice compared to our ten minutes. However, the training regimen, 45 min or 20 min FOI vs. ten minutes, differed from ours [18,31].

We focused on a small part (agility) of the procedure of performing an awake FOI, whereas previous studies considered the entire procedure [9,12,26]. As we did not practice and pre-test airway anatomy knowledge, we might have overestimated participants’ ability to properly recognise the altered anatomy of the airway, which could have had an impact on the time to reach the carina [21,31,32,33].

Another limitation of our study was its single-centre design. Although our department has a very low threshold for performing an awake FOI in general, due to the low incidence of Head and Neck surgery at our hospital, awake FOI is practiced less frequently; therefore, the individual overall number of FOIs performed was low. This overall low level of exposure to FOI led to a small expert group based on the division of the group on normal clinical practice and seniority with the group of anaesthetists seen as experts, the residents as intermediate, and the anaesthesia nurses as novices. Recommendations in the literature on the number of procedures necessary to obtain proficiency for bronchoscopy range from twenty-five up to one hundred procedures [7,8,9,10,11,34]. However, only 10% of the 72 participants, and only about half of the expert group had performed more than twenty-five awake FOIs, probably indicating a lack of extensive experience even in consultant anaesthetists.

Medical simulation has some inherent limitations, such as the inadequate imitation of human systems, flawed learning due to a lack of signs and signals, cost and technical issues, etc. [35,36]. This means that the fact that our study was conducted exclusively on a simulator in a controlled environment can also be considered a limitation, as there is little evidence to support the transfer of simulation-based learning to the clinical setting.

The strengths of our study include the use of different scenarios at different time points to prevent the recall of the simulated scenarios. Randomization between the intervention and control groups was performed, and the baseline characteristics of the participants showed no significant differences between the groups. The use of an objective scoring system, the intrinsic automated scoring generated by the bronchoscopy simulator, which compared to the more-subjective scoring method by independent assessors, is another strength of our study.

## 5. Conclusions

In summary, although there is evidence that practice on simulators improves the performance of an FOI in a normal airway, we were unable to show a training effect on a difficult airway. We need to further investigate the different training aspects to design a proper curriculum for improving the performance of an awake FOI, especially in patients with a difficult airway.

## Figures and Tables

**Table 1:** Baseline characteristics.

	Control	Intervention	*p*-Value
*n*	33	35	
Sex = female (%)	18 (54.5)	11 (31.4)	0.085
Age in years (mean (SD))	38.85 (9.97)	38.91 (10.52)	0.838
Experience in anaesthesia in years (mean (SD))	10.70 (9.99)	10.60 (9.95)	0.964
Function, *n* (mean (SD))			0.984
Anaesthesia resident	12 (36.4)	12 (34.3)	
Anaesthesia consultant	11 (33.3)	12 (34.3)	
Anaesthesia nurse	10 (30.3)	11 (31.4)	
Number of participants having completed x years of residency, *n* (%)			0.956
1st/2nd year	4 (33.3)	4 (33.3)	
3rd/4th year	6 (50.0)	5 (41.7)	
5th year/fellows	2 (16.7)	3 (25.0)	
Number of participants having completed x FOI * in the last 12 months, *n* (%)			0.268
0–3x	29 (87.9)	29 (82.9)	
3–8x	1 (3.0)	5 (14.3)	
8–15x	2 (6.1)	1 (2.9)	
15–25x	0 (0.0)	0 (0.0)	
25–50x	1 (3.0)	0 (0.0)	
>50x	0 (0.0)	0 (0.0)	
Number of participants having experience with Flexible Scope outside anaesthesia, *n* (%)			0.487
0x	21 (63.6)	23 (65.7)	
1–10x	6 (18.2)	9 (25.7)	
11–25x	4 (12.1)	1 (2.9)	
25–50x	0 (0.0)	0 (0.0)	
>50x	2 (6.1)	2 (5.7)	
Number of participants having completed x type of training, *n* (%)			
Specific airway course = yes (%)	7 (21.2)	9 (25.7)	778
Workshop during a conference = yes (%)	4 (12.1)	5 (14.3)	0.792
On the job training = yes (%)	19 (57.6)	20 (57.1)	0.971
Training at the ENT ** department = yes (%)	6 (18.2)	6 (17.1)	0.911
Training at the pulmonology department = yes (%)	2 (6.1)	5 (14.3)	0.429
No training at all = yes (%)	11 (33.3)	14 (40.0)	0.621

Abbreviations: * FOI = Fibreoptic intubation, ** ENT = Ear, Nose and Throat Department.

**Table 2:** Results of questionnaire.

	Whole Group	Control	Intervention
*n*	68	33	35
Experience in Anaesthesia at baseline (median (IQR))	6.25 (3.0–16.25)	6.0 (3.0–17.5)	7.0 (3.0–14.0)
Number of participants having completed x FOI at baseline, *n* (%)			
0–3x	34 (50)	17 (51.5)	17 (48.6)
3–8x	13 (19.1)	5 (15.2)	8 (22.9)
8–15x	4 (5.9)	2 (6.1)	2 (5.7)
15–25x	4 (5.9)	4 (12.1)	0 (0.0)
25–50x	7 (10.3)	2 (6.1)	5 (14.3)
>50x	6 (8.8)	3 (9.1)	3 (8.6)
Number of participants having completed x FOI at 12 months, *n* (%)			
0–3x	58 (85.3)	29 (87.9)	29 (82.9)
4–8x	6 (8.8)	1 (3.0)	5 (14.3)
8–15x	3 (4.4)	2 (6.1)	1 (2.9)
15–25x	0	0	0
25–50x	1 (1.5)	1 (3.0)	0
>50x	0	0	0
Number of participants having completed x FOI in last 12 months before study participation, *n* (%)			
0–3x	31 (45.6)	29 (87.9)	29 (82.9)
3–8x	13 (19.1)	1 (3.0)	5 (14.3)
8–15x	4 (5.9)	2 (6.1)	1 (2.9)
15–25x	5 (7.4)	0 (0.0)	0 (0.0)
25–50x	6 (8.8)	1 (3.0)	0 (0.0)
>50x	8 (11.8)	0 (0.0)	0 (0.0)
Number of participants having completed x FOI during study participation at 12 months, *n* (%)			
0–3x	58 (85.3)	29 (88)	29 (82.9)
3–8x	8 (11.7)	3 (9)	5 (14.3)
8–15x	1 (1.5)	0	1 (2.9)
15–25x	0	0	0
25–50x	1 (1.5)	1 (3)	0
>50x	0	0	0
Estimation of own skills at baseline	23 (0–65.8)	20 (0.0–68.0)	37 (0.0–65.0)
(median (IQR))			
Estimation of own skills at 12 months (median (IQR))	53 (17.0–70.0)	52 (18.5–71)	56 (15.5–70.3)
Estimation of stress level at baseline (median (IQR))	43.5 (30.0–43.5)	42 (30.0–60.5)	44 (25.0–68.0)
Estimation of stress level at 12 months (median (IQR))	48 (24.0–65.0)	41 (20.5–60.0)	56 (25.5–70.0)
Confidence in own FOI performance at baseline, *n* (%)			
yes	33 (48.5)	16 (48.5)	19 (57.6)
no	35 (51.5)	17 (51.5)	14 (42.2)
Confidence in own FOI performance at 12 months, *n* (%) *			
yes	44 (64.7)	17 (48.6)	25 (71.4)
no	23 (33.8)	18 (51.4)	9 (25.7)

Abbreviations: * One missing in intervention (*n* = 34).

## Data Availability

The data presented in this study are available on duly request from the corresponding author.
